# Virome of a Feline Outbreak of Diarrhea and Vomiting Includes Bocaviruses and a Novel Chapparvovirus

**DOI:** 10.3390/v12050506

**Published:** 2020-05-04

**Authors:** Yanpeng Li, Emilia Gordon, Amanda Idle, Eda Altan, M. Alexis Seguin, Marko Estrada, Xutao Deng, Eric Delwart

**Affiliations:** 1 Vitalant Research Institute, 270 Masonic Avenue, San Francisco, CA 94118, USA; alphaleeyp@hotmail.com (Y.L.); EAltan@vitalant.org (E.A.); xdeng@vitalant.org (X.D.); 2 Department of Laboratory Medicine, University of California, San Francisco, CA 94118, USA; 3 The British Columbia Society for the Prevention of Cruelty to Animals, Vancouver, BC V5T1R1, Canada; egordon@spca.bc.ca (E.G.); midle@spca.bc.ca (A.I.); 4 IDEXX Reference Laboratories, Inc., West Sacramento, CA 95605, USA; seguin@idexx.com (M.A.S.); Marko-Estrada@idexx.com (M.E.)

**Keywords:** *Parvoviridae*, *chaphamaparvovirus*, chapparvovirus, fechavirus, diarrhea, virome

## Abstract

An unexplained outbreak of feline diarrhea and vomiting, negative for common enteric viral and bacterial pathogens, was subjected to viral metagenomics and PCR. We characterized from fecal samples the genome of a novel chapparvovirus we named fechavirus that was shed by 8/17 affected cats and identified three different feline bocaviruses shed by 9/17 cats. Also detected were nucleic acids from attenuated vaccine viruses, members of the normal feline virome, viruses found in only one or two cases, and viruses likely derived from ingested food products. Epidemiological investigation of disease signs, time of onset, and transfers of affected cats between three facilities support a possible role for this new chapparvovirus in a highly contagious feline diarrhea and vomiting disease.

## 1. Introduction

Cats have an estimated world-wide population of over half a billion. Members of at least 15 viral families have been found to infect cats, including rabies virus, feline rotavirus (FRV), feline panleukopenia virus (FPV), feline bocaviruses (FBoV), feline bufavirus (FBuV) [1], feline astroviruses (FeAstV), feline picornaviruses (FePV), feline enteric coronavirus (FECV), feline calicivirus (FCV), feline herpesvirus (FHV-1), feline immunodeficiency virus (FIV) and feline leukemia virus (FeLV) [2,3,4]. Diarrhea in cats is common and possible infectious causes include bacteria, parasites, and/or viruses. Some of the most prevalent feline enteric viruses include FBoV, FeAstV, FRV, and FPV [3,5,6]. Conditions in animal shelters contribute to pathogen emergence due to factors including intensive housing, rapid population turnover, animal stress, and the presence of many direct and indirect routes of possible exposure [2].

The *Parvoviridae* family consists of non-enveloped, icosahedral viruses with single-stranded DNA genomes of 4 to 6 Kb [7,8]. Eight ICTV-approved genera are currently included in the *Parvoviridae* family [9]. A recent reorganization of the *Parvoviridae* has proposed the creation of a third subfamily named *Hamaparvovirinae* that includes both invertebrate and vertebrate viruses as well as endogenized genomes in the germ lines of fish and invertebrates, suggesting an ancient origin [10,11]. Vertebrate infecting members of this subfamily are classified within the *Chaphamaparvovirus* genus (previously labeled as unclassified or chapparvoviruses) and have been identified in rats and mice [12,13], bats [14,15], rhesus macaques [16], cynomologus macaques [17], dogs [18], pigs [19], Tasmanian devils [20], birds (red-crowned crane [21], chicken [22], and turkey [23]) and fish (Tilapia [24]). A chapparvovirus has also been reported in a Gulf pipefish and has been proposed to belong to a second genus named *Ichthamaparvovirus* [10]. A recent study showed that a murine chaphamaparvovirus named murine kidney parvovirus (MKPV) was the cause of nephropathy in laboratory mice [25] and was widely distributed world-wide [26]. 

Here, we analyzed a multi-facility outbreak of vomiting and diarrhea in cats using the following approaches: a commercial feline diarrhea panel of PCR tests for known enteric pathogens; viral metagenomics; and follow-up PCRs. Multiple mammalian viruses of varied origins were detected. Diverse feline bocaviruses and a novel chaphamaparvovirus we named fechavirus were each shed by approximately half of the affected animals tested. The epidemiology of the outbreak points to a possible role for this newly characterized parvovirus in feline vomiting and diarrhea, pending larger studies. 

## 2. Materials and Methods

### 2.1. Sample Collection and Pathogen Screening

From November 2018 to January 2019, a multi-facility outbreak of feline vomiting and diarrhea occurred in an animal shelter system in British Columbia, Canada. A case definition was created to identify all animals affected by the outbreak while excluding unaffected animals: cats housed in three affected facilities with one or more episodes of vomiting or diarrhea (with no apparent other cause) and direct or indirect exposure to cats from the case, between 8 November 2018 and 28 January 2019 (supplementary Table S1). Direct exposure was defined as being housed communally with possible direct contact with feces, vomit or a body surface of another animal meeting the case definition (based on time and animal location). Indirect exposure was defined as exposure via fomites, personnel, or an environment potentially contaminated by another animal meeting the case definition. A total of 43 cats met the case definition. An outbreak investigation was performed. Stool samples from 27 sick cats (21 individuals, two pooled litters of 3 co-housed kittens each) were collected and submitted for a variety of diagnostic tests (see supplementary Table S2). As animal shelters are resource-limited, testing was focused on clinically sick cats and no samples were collected from healthy cats. Initial diagnostic testing included fecal flotation (for parasites) (12 cats), fecal antigen testing (helminth (9 cats), *Giardia* (9 cats), and FPV (3 cats)). These tests did not yield a causative agent that could explain the outbreak.

Testing was then expanded and 12 fecal samples representing a subset of 14 cats (11 individuals, one pooled sample from a litter of 3 kittens) were submitted to IDEXX Laboratories, Inc (Sacramento, CA, USA) and subjected to a comprehensive feline multi-pathogen diarrhea screening panel [27]. This panel tested for *Clostridium perfringens* alpha toxin, *Clostridium perfringens* enterotoxin, *Campylobacter coli* and *Campylobacter jejuni*, *Cryptosporidium spp*., *Giardia spp*., *Salmonella spp*., *Tritrichomonas foetus*, *Toxoplasma gondii*, FPV, FECV, and FRV using PCR and RT-PCR. This screening also did not yield a causative agent that could explain the outbreak. Most samples were collected within five days (only 2 were within 10 days) after onset of vomiting or diarrhea with treatments generally initiated after sample collection. Four feline vomit samples were also collected from a room of cats in the third affected shelter between January 20 and 28, 2019, but it was not possible to identify which cats had produced these samples. 

Once the unusual nature of the outbreak became evident (indirect transmission pattern and large number of animals involved), any feces that was not discarded during the initial round of diagnostics and a small number of serially collected samples were frozen for possible further analysis. Only 17 initial samples from 19 sick cats (16 individuals and a pooled litter of 3 kittens) were available for analysis. Serial samples (collected on more than one day) were available from 4 of these individual cats.

### 2.2. Viral Metagenomics Analysis

One gram of feces from each of the cats was vortexed in 2 mL phosphate buffer saline (PBS) with zirconia beads. Viral particles were then enriched according to previously described methods [28] by filtration through a 0.45 µm filter (Merck Millipore, Massachusetts, USA) and digestion of the filtrate was achieved with a mixture of nuclease enzymes prior to nucleic acid extraction. Nucleic acids (both RNA and DNA) were then amplified using random RT-PCR as described [28]. Briefly, reverse transcription was performed with primer with a random nonamer at the 3’ end (5’GCCGACTAATGCGTAGTCNNNNNNNNN), followed by second strand synthesis using Klenow Fragment DNA polymerase (New England Biolabs, Massachusetts, USA). Both cDNA and DNA were then amplified by AmpliTaq Gold DNA polymerase (Thermo Fisher Scientific, Massachusetts, USA) using a primer consisting of the fixed portion of the random nonamer containing primer (5’GCCGACTAATGCGTAGTC). ssDNA genomes are converted to dsDNA during the Klenow DNA polymerase primer extension step. An Illumina library was then generated using the transposon-based Nextera XT Sample Preparation Kit and sequenced on the MiSeq platform (2 × 250 bases, dual barcoding) (Illumina, CA, USA). Adaptor and primer sequences are trimmed using the default parameters of VecScreen which is part of BLAST package v2.2.31 [29]. Reads are considered duplicates if base positions 5 to 55 are identical. One random copy of duplicates is kept. Low sequencing quality tails are trimmed using Phred quality score 20 as the threshold. Human and bacterial reads were subtracted by mapping to human reference genome hg38 and bacterial nucleotide sequences from BLAST NT database using Bowtie2 v2.2.4 [30]. The Ensemble Assembler program (v1.0) [31] was used for de novo assembly. Both contigs and singlets were then analyzed using the BLASTx (v.2.2.7) to in-house viral proteome database, then the significant hits to virus are aligned to BLAST NR universal proteome database using DIAMOND v0.9.15.116 [32]. The short reads sequencing data are available at NCBI Sequence Read Archive (SRA) under the BioProject number PRJNA565775 (Biosample accession SAMN13526805-13526821, and SAMN13672796).

### 2.3. Genome Assembly of Novel Chaphamaparvovirus

Geneious R11 program was used to align reads and contigs to reference viral genomes and generate partial genome sequence. The genome gaps from the initial assembly were then filled by PCR whose products were Sanger sequenced.

### 2.4. Diagnostic PCR and Prevalence

DNA was extracted from each individual fecal sample (and one pool of 3, cat#973–975) shown in Table 3 plus 4 vomit samples using the QIAamp MinElute Virus Spin Kit (Qiagen, Hilden, Germany), and PCR assays were used for the detection of different viral nucleic acids in each sample. For fechavirus, the first round PCR primers FechaF1 (5’-GGTGCGACGACGGAAGATAT-3’) and FechaR1 (5’-CAACACCACCATCTCCTGCT-3’) amplified a 332bp region. The second round of primers: FechaF2 (5’-GCTGCAGTTCAGGTAGCTCA-3’) and FechaR1 amplified a 310 bp region. The PCR conditions for both rounds are as follows: 1× PCR Gold buffer I, 0.2mM dNTPs, 0.4µM of each forward and reverse primers, 1U of Amplitaq Gold DNA polymerase (Applied Biosystems, MA, USA) and 2 µL DNA template in a final 25 µL reaction. The PCR programs for both rounds: 95 °C 5 min, 35 cycles for 95 °C 15 s, 58 °C 30 s and 72 °C 30 s, followed by an extension at 72 °C for 7 min. PCR products were verified by gel electrophoresis and Sanger sequencing. The PCR primers amplifying all three feline bocaviruses are as follows: FBoV-F 5’-AGAACCRCCRATCACARTCCACT-’3 and FBoV-R 5’-TGGCRACCGCYAGCATTT CA-’3, the PCR conditions are the same as described before [33]. The primers for FCV are: calici-F 5’-GCAAAGGTGGCGTCAAACAT-’3 and calici-R 5’-GCAAAGGTGGCGTCAAACAT-’3. The PCR programs: 95 °C 5 min, 40 cycles for 95 °C 30 s, 55 °C 40 s and 72 °C 40 s, followed by an extension at 72 °C for 10 min.

### 2.5. Phylogenetic Analysis

The NS1 and VP1 protein sequences were aligned using the Muscle program in MEGA 7.0; phylogenetic trees were inferenced using Maximum-Likelihood method. Model test module imbedded in MEGA was used to determine the best substitution model. Phylogenetic trees of both NS1 and VP1 protein sequences were generated using the bootstrap method under GTR+I+G model.

### 2.6. Ethics Statement

The authors confirm that the ethical policies of the journal, as noted on the journal’s author guidelines, have been adhered to. No ethical approval was required as samples were collected as part of routine outbreak investigation.

### 2.7. Data Availability Statement

The data that supports the findings of this study are available in the main body of this article.

## 3. Results

### 3.1. Epidemic and Clinical Data

A vomiting and diarrhea outbreak was identified across three animal shelters in British Columbia, Canada, lasting from November 2018 to January 2019. A total of 43 cats met the case definition (see methods). Seventeen samples from 19 cats (including a pool of samples from three cats), plus four vomit samples, were available for further study. 

**Figure 1:**
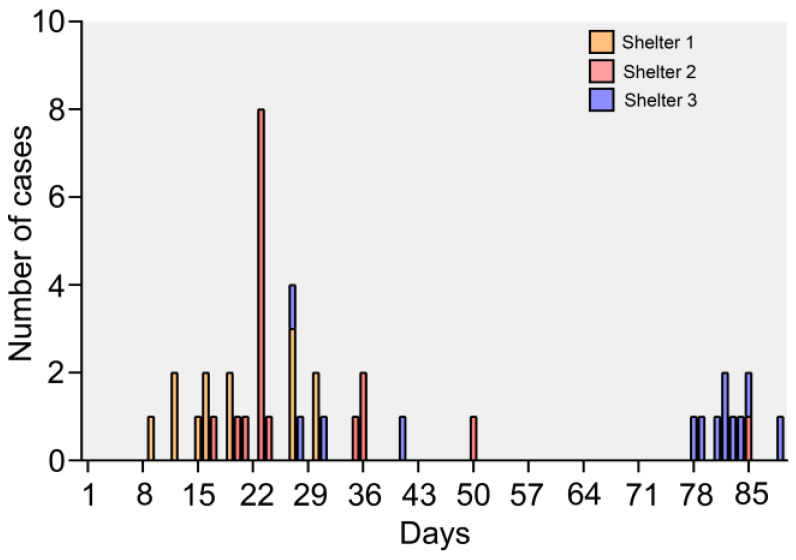
Epidemic curve showing dates of illness onset for cat vomiting and diarrhea cases in outbreak in three shelters from November to January.

**Figure 2:**
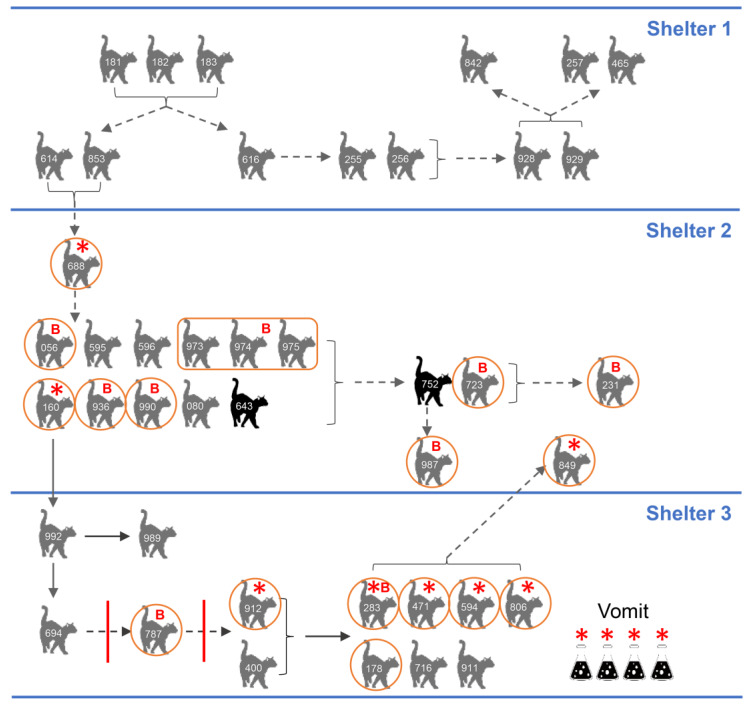
Outbreak diagram in three shelters. Animal ID shown for each cat (also see Supplementary Tables). Grey cats: clinically affected and recovered; black cats were euthanized. Red circle around a cat means feces was sampled individually; rectangle around the three cats means a litter of kittens were sampled together. Solid arrow means direct or indirect contact possible (housed communally), dotted line means indirect contact only. Bracket indicates group of affected animals with transmission to another group. Red solid vertical line indicates failed clean break. Red star indicates fechavirus PCR positive cats/samples. Red letter “B” means FeBoV PCR positive cats/samples.

The outbreak was first identified on November 24, 2018 in Shelter 2 when 8/12 cats housed in the main adoption room became sick with vomiting and diarrhea. Diet, environmental, and toxic causes of the outbreak were ruled out clinically and an outbreak investigation initiated. The first cases in Shelter 2 (#614, #853) had arrived from Shelter 1 via the organization’s animal transfer program on November 15, 2018 and were sick during or shortly after transport. Upon further investigation, the originating shelter, Shelter 1, had 11 more sick cats. On November 20, Shelter 3 became involved in the outbreak when a cat (#160) transferred from Shelter 2 became sick, several days after arrival and before the outbreak had been identified. Ultimately, a total of 13 cats were affected in Shelter 1 in November, 17 cats were affected in Shelter 2 (November–January), and 13 cats were affected in Shelter 3 (November-January) (Figure 1 and Supplementary Table S1). Nearly all transmissions were indirect (Figure 2). Because of this, it was not possible to definitively determine which animals had been exposed, except in specific rooms where housing was communal or exposure was known to be widespread prior to the introduction of control measures. Attack rates (number of sick animals/total number of animals in a defined population) for these rooms were 66.7% (Shelter 2) and 83.3% (Shelter 3) (Table 1).

Overall, diarrhea and vomiting were observed in 81.4% and 67.4% of the 43 cases, respectively. There were likely more cats vomiting because vomitus that could not be attributed to a particular cat was found multiple times in the final wave of illness. Of affected cats, 25.6% and 11.6%, respectively, also showed inappetence and lethargy, and 67.4% required veterinary care (Table 2). The minimum incubation period was 24 h, and the maximum was estimated at 5–7 days based on estimated exposure dates. Vomiting tended to start 1–2 days before diarrhea and last only a couple of days, but in some animals, the diarrhea lasted up to a week (longer in a few animals). The mean duration of illness was 5.1 days, with a median of 4.0 days (range 1 to 19 days) (Supplementary Table S1). No recovered cats relapsed and there were no cases where transmission was traced to a clinically recovered animal.

The sheltering organization initiated control measures on 24 November 2018 including cessation of all cat movement, use of personal protective equipment (gowns, gloves, caps, shoe covers) in all cat housing areas, and enhanced sanitation measures using accelerated hydrogen peroxide, which has good efficacy against bacteria and viruses (including non-enveloped viruses) [34]. After control measures were initiated, the outbreak slowed and cases became sporadic, except for at Shelter 3 in January (due to communal housing). Each time control measures failed, the failure was traced to a contaminated environment or fomite. 

### 3.2. PCR Pathogen Screening 

Seventeen fecal samples from cats in Shelters 2 and 3 (collected after the outbreak was recognized) were available for further analysis. Twelve samples representing 14 cats were subjected to a comprehensive feline diarrhea panel pathogen screen (see Materials and Methods). FECV was detected in one sample, *Giardia* DNA in three samples, and *Clostridium perfringens* alpha toxin DNA in 6 out of 12 samples, while *Clostridium perfringens* enterotoxin results were negative for all animals. *Clostridium perfringens* is part of the normal feline intestinal flora; diarrheic and clinically healthy cats shed the organism at similar rates [35]. Type A, the most commonly isolated biotype, produces alpha-toxin and is commonly detected in feces of both diarrheic and healthy dogs [36]. It is not considered a primary cause of diarrhea in cats, but may contribute to diarrhea in cases where a disturbance in the intestinal microenvironment has occurred, such as due to concurrent pathogen infection [37]. *Giardia* is a protozoal parasite that can infect multiple mammalian species and can be associated with diarrhea (and rarely, vomiting) in cats [38]. It has a prepatent period of 5–16 days and while it can cause shelter outbreaks, it would not be capable of causing disease only 24 h after exposure [38]; a shelter outbreak of *Giardia* would have a more indolent course. FRV was negative for all samples tested, only one sample (from a pooled litter) was positive for FPV. These tests ruled out the primary bacterial differentials for a shelter outbreak with a short incubation period. None of the bacteria/viruses/parasites testing positive were considered the main cause of the vomiting and diarrhea outbreak. 

### 3.3. Viral Metagenomics Analysis

In order to identify pathogens that could have caused the outbreak, all 17 available fecal samples were analyzed using viral metagenomics. Viral sequences assigned to five main viral families (*Anelloviridae, Parvoviridae, Papillomaviridae, Polyomaviridae* and *Caliciviridae*) were detected in these fecal samples (Table 3). 

### 3.4. Anelloviruses, Papillomaviruses and Polyomavirus

Feline anellovirus reads were detected in three cats, and unclassified anellovirus reads were detected in another four cats. Anelloviruses are widely prevalent in humans and other mammals, and no clear pathologic role of anelloviruses has been identified [39,40]. Lyon-IARC polyomavirus and feline papillomavirus (*Dyothetapappillomavirus* 1) were shed by one and two cats, respectively, with both genomes showing nucleotide identity of ~99% with those genomes previously reported in cats [5,41]. Lyon-IARC DNA was previously shown to be shed by 3/5 cat feces in a hoarding situation diarrhea outbreak (one of which was co-infected with FPV) [5,41]. 

### 3.5. Dietary Contamination 

Chicken anemia virus (CAV), and gyrovirus 4 and 6 DNA from family *Anelloviridae* were each detected in one cat. CAV is a highly prevalent chicken pathogen [42] and gyroviruses have been frequently detected in human feces presumably originating from consumed infected chicken. [43,44]. Also detected were two sequence reads with a perfect match to porcine parvovirus 5 (*Protoparvovirus* genus) also likely from consumed food [45]. 

### 3.6. Vaccine-Derived Sequences

All cats were vaccinated subcutaneously upon entry into the three facilities with Felocell 3 vaccine (Zoetis Inc., Parsippany, NJ, USA) containing live attenuated FPV, FCV (mainly associated with the respiratory system in cats [46]), and feline herpesvirus-1. The vaccine was also sequenced using metagenomics, yielding the near complete FCV genome (deposited in GenBank MN868063), approximately 85.8% of the FPV genome, and more than 20,000 reads of the herpes virus. Felocell 3 vaccine-derived FPV and FCV sequences were compared to those detected by metagenomics in two and four of the diarrheic cats respectively. The sequence reads in the two cats shedding FPV showed 99%–100% identity to the vaccine sequences. The few reads of FCV (1 to 4 sequence reads per sample) also showed a high level of similarity (99–100%) to the sequenced vaccine FCV genome. Given the extensive sequence diversity of FPV and feline FCV [47,48,49,50], we conclude that the FPV and FCV sequence reads detected in feces by metagenomics were vaccine derived. 

### 3.7. Parvoviruses

Viral sequences belonging to the *Bocaparvovirus* and *Chaphamaparvovirus* genera were the most abundant and detected in 14/17 samples (Table 3). Three different feline bocaparvoviruses (FeBoV1-3) were found in 3, 3 and 2 samples, respectively. We then used a pan-FeBoV PCR to test these 17 samples. One cat, FeBoV negative by metagenomics, was PCR positive (FeBoV2 by Sanger sequencing of PCR amplicon). Two other cats positive for bocaviruses with only 2 sequence reads were negative by PCR. All other PCR results were consistent with metagenomics results (Table 3). All together, FeBoV DNA was detected by metagenomics and/or PCR in 9/17 samples.

**Figure 3:**
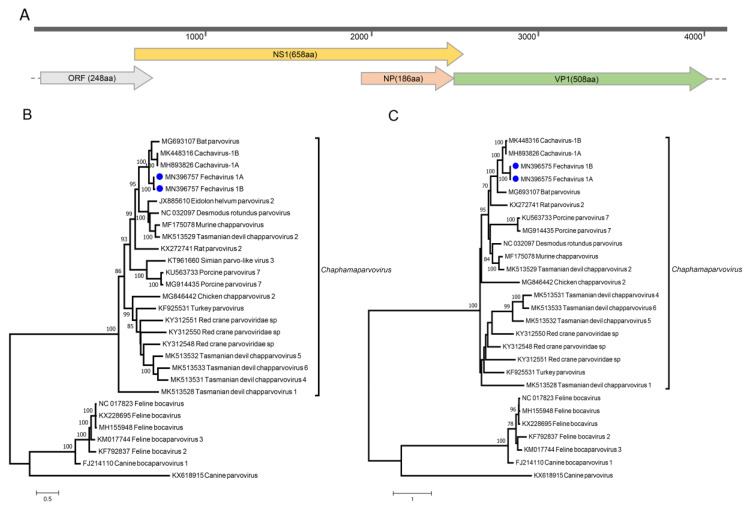
(**A**) Genome organization of fechavirus. Maximum-likelihood phylogenetic trees of NS1 protein sequences (**B**) and VP1 protein sequences (**C**). Fechaviruses found in this study were highlighted with blue cycles.

Six cats also yielded sequence reads related to multiple parvoviruses in the *Chaphamaparvovirus* genus. Using de novo assembly and specific PCR to fill gaps, two near full-length genomes of 4225 and 4134 bases (Figure 3A) were generated from cats #594 and #849 (GenBank accession numbers MN396757 and MN794869). The two genomes share 99.1% identity of NS1 and 99.6% identity of VP1 at protein level, and 99.2% and 99.3% at nucleotide level. These genomes encoded the two main ORFs shared by all parvoviruses, a 658aa ORF encoding non-structural replication protein (NS1), and a 508aa ORF encoding viral capsid (VP1). The sequence of the inverted terminal repeats was not completed. The closest relative to these genomes was a parvovirus recently reported in canine feces named cachavirus (MK448316) [18], with a nucleotide identity of 74.2% and NS1 and VP1 protein identity of 65.5% and 68.8%, respectively. Other major ORFs consisted of a 186aa ORF within the NS1 labeled NP and a potential 248aa ORF partially overlapping the 5’ of the NS1 ORF. The NP ORF is widely conserved in chaphamaparvoviruses [25,26]. No significant similarity was found for the 248aa ORF.

Under an April 2019 ICTV proposal updating the latest published classification [9], members of the same parvovirus species should show >85% identity for NS1. The fechavirus genome, therefore, qualifies as a member of a new species in the proposed *Chaphamaparvovirus* genus. We named this novel virus feline chaphamaparvovirus or fechavirus. Phylogenetic analysis confirmed the closest relative to be the canine cachavirus in both NS and VP ORFs (Figure 3B,C).

Specific PCR primers were designed based on the fechavirus genomes and used to test all 17 fecal samples. Besides the six fechavirus-positive fecal samples detected by NGS, two more samples were positive by PCR. All four vomit samples tested (from unknown cats in Shelter 3) were also PCR positive. A total of eight animals therefore shed fechavirus, of which one was co-infected with FeBoV3 (Cat #283). Of the 17 samples available for analysis, only one was negative for both FeBoV and fechavirus DNA (cat # 178).

Longitudinally collected fecal samples were available from four cats and PCR tested for fechavirus DNA. Cat #688 was fechavirus-positive on the last day of his illness (first available sample). Cats #912 and #283 were each positive at only a single time point but manifested disease signs for 9 and 4 more days, respectively. Cat #594 shed fechavirus over 4 days while symptomatic plus a further 7 days after recovery (Table 4).

**Figure 4:**
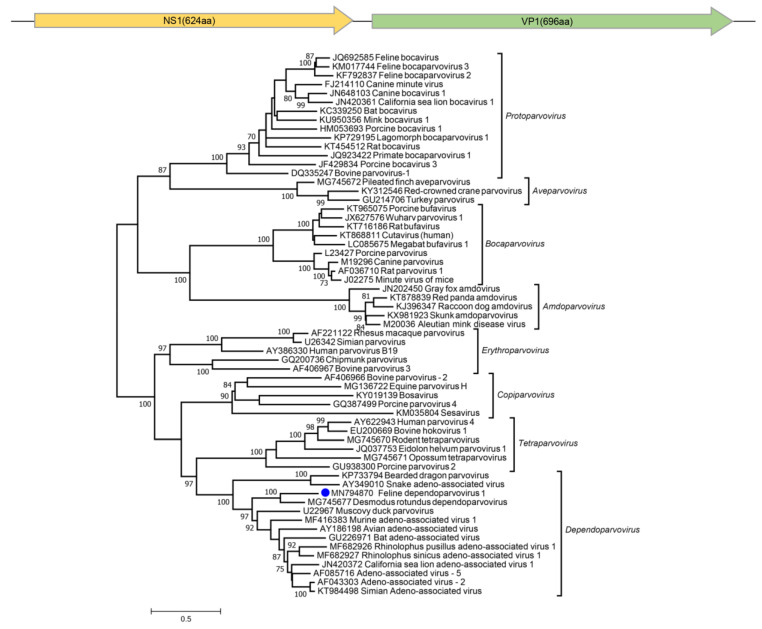
Two main ORFs of the new feline dependoparvovirus and maximum-likelihood phylogenetic tree of the complete NS1 protein sequences of subfamily *Parvovirinae*. The new dependoparvovirus identified in cat is shown with a blue circle.

Dependoparvovirus sequence reads were also found in two cats. A near full-length genome of 4315 bases could be assembled from cat #849 (also infected with fechavirus) whose phylogenetic analysis showed it to be related to a recently reported dependoparvovirus genome from bats (*Desmodus rotundus*) sharing NS1 and VP1 protein with 51.6% and 46.7% identities (Figure 4). 

### 3.8. Epidemiology

The timeline of disease presentation and fecal shedding status of key animals in the transmission chain between the three facilities was determined. There were no samples from cats in Shelter 1 available for testing, but cat #688 became sick and was fechavirus-positive shortly after being exposed to cats #853 and #614, who originated in Shelter 1 and became sick during transfer to Shelter 2. Cat #688 entered Shelter 2 on November 14, 2018. Cats #853 and #614 were transferred to Shelter 2 on November 15, and cat #688 became sick on November 17. Of the other 16 affected cats in Shelter 2 a total of five individual cats and a sample pool (mixed feces from three cats) were FeBoV1-, 2-, or 3-positive. A second cat in Shelter 2 was shedding fechavirus (cat #160). This cat was transferred to Shelter 3, where he was in direct contact with subsequently sick cat #992, who was then in contact with cats #989 and #694, initiating an illness transmission chain in Shelter 3. Despite clean breaks imposed by cleaning and emptying the facility in Shelter 3, illness continued to recur and spread. The most likely cause of this was fomites that had been touched by humans caring for the cats, but were not in the cat housing area and had not been thoroughly disinfected; the outbreak resolved once this was addressed. Of the seven cat fecal samples available for study from Shelter 3, fechavirus was detected in five and bocaparvovirus in two animals. Only a single case yielded both fechavirus and bocaparvovirus DNA (#283). Four additional vomit samples from unknown cat(s) in Shelter 3 were all fechavirus PCR-positive. Last, an initially healthy cat in Shelter 2 developed disease signs immediately after cats from Shelter 3 were returned to Shelter 2 during the last wave of the outbreak and was found to be fechavirus-positive (cat #849). 

## 4. Discussion

Currently identified feline parvoviruses belong to two genera of the *Parvoviridae* family, namely three bocaparvovirus species (*Carnivore bocaparvovirus 3/4/5*, which include FeBoV1-3 [5,51,52]), and two protoparvovirus species (*Carnivore protoparvovirus 1* which includes FPV [53] and the still unclassified FBuV which is closely related to canine bufavirus [1]). FeBoV1 was first discovered in multiple tissues of cats in Hong Kong [53] and subsequently reported in cat feces in the US, Japan, Europe and China [3,5,51,52]. Similar to human bocaparvovirus DNA commonly found in the feces of healthy humans [54,55,56], frequent feline bocaparvovirus DNA detection in healthy cats raises questions regarding its pathogenicity in cats [1,3,5,53,57]. A recent study showed a possible association between FBoV-1 infection, potentially aggravated by FPV, and hemorrhagic enteritis [1,3,5,53,57]. FPV is an extensively studied pathogen that can lead to reduction in circulating white blood cells and enteritis [58,59]. The pathogenicity of FeBuV in cats is currently unknown [1,60] but the virus shares very high sequence identity with a canine bufavirus [1,61,62].

Using metagenomics, we found FeBoV1, 2, and 3 and a novel chaphamaparvovirus we named fechavirus in a large fraction of fecal samples and fechavirus in all vomit samples from sick cats in a multi-facility outbreak. Subsequent PCR testing confirmed the presence of either a bocavirus or fechavirus or both (*n* = 1 cat #283) of these viruses in all but one of the 17 samples tested (cat #178). The outbreak in Shelter 2 predominantly tested positive for FeBoV (7/9 FeBoV +ve and 2/9 fechavirus +ve), while the sick cats in Shelter 3 were mainly shedding fechavirus (6/7 fechavirus +ve, 2/7 FeBov +ve). Another cat housed in Shelter 2 who developed vomiting after sick cats were transferred back from Shelter 3 was also shedding fechavirus as well as a novel dependovirus. Many although not all dependoviruses are replication-defective and require a helper virus [63]. The co-detection of fechavirus may provide the required help for replication of this new feline dependovirus. 

FPV and feline FCV reads were determined to originate from recently inoculated attenuated vaccine strains, while other viruses were deemed to be asymptomatic infections, derived from chicken and pork viruses in consumed food, or detected only in sporadic cases. FCV sequences were negative by PCR. This could be the result of low viral loads in those cats.

The close genetic relationship of fechavirus to cachavirus found in both diarrheic and healthy dog feces [18] may reflect a cross-carnivore virus transmission as occurred with FPV mutating into the highly pathogenic canine parvovirus [64,65].

Serially collected fecal samples from symptomatic cats revealed transient shedding for 3/4 animals with fechavirus DNA only detected at a single time point. One animal shed fechavirus DNA for 12 days both while exhibiting disease signs and 7 days after disease resolution. The short duration of fechavirus shedding for 3/4 animals, also typical of FPV shedding [66], may account for its non-detection in some of the affected animals in Shelter 2 and 3. All but one of the fechavirus-negative samples were positive for one of three different feline bocaviruses indicating that both bocaviruses and fechavirus were circulating in these shelters. Because of the high diversity of bocaviruses found here, belonging to three distinct species, and the typically asymptomatic nature of FeBoV infections, a role for bocaviruses in the multi-facility transmission of this disease outbreak seems unlikely. A pathogenic role for fechavirus seems more likely as viral DNA was detected in key animals with contacts across the three facilities experiencing outbreaks with similar disease signs. 

A key clinical attribute of this unusual outbreak was the prominence of indirect routes of transmission, including ongoing fomite transmission after robust control measures were implemented. Another notable characteristic of this case was the very short minimum incubation period. Combined with the absence of any known pathogen identified on routine screening, this points to a novel non-enveloped enteric virus as the most likely causative agent. Animal shelters and veterinary hospitals should maintain good routine infection control procedures and consider novel viruses, including fechavirus, in the diagnosis of feline gastrointestinal disease.

In this study, we characterized the virome from a cat diarrhea and vomiting outbreak, and showed that besides different FeBoV, a new chaphamaparvovirus was associated with gastrointestinal disease. Future studies on its prevalence, genetic diversity, and tissue distribution in healthy and diarrheic cats are needed to further investigate a possible etiologic role in feline disease.

## Figures and Tables

**Table 1:** Attack rates (AR) for selected rooms and dates where widespread or complete exposure was suspected.

	Exposed (Presumptive)			
Location	Sick	Not Sick	Total	AR%
Shelter 2 *	8	4	12	66.7%
Shelter 3 **	10	2	12	83.3%

* 23 November 2018: Main adoption room; ** 6 December 2018-23 January 2019: Communal cat area.

**Table 2:** Summary of clinical signs and need for outside veterinary care for 43 cats meeting case definition.

Clinical Signs	#of Cats (*n* = 43)	Percentage
Diarrhea	35	81.4%
Vomiting	29	67.4%
Inappetence	11	25.6%
Lethargy	5	11.6%
Required veterinary visit	29	67.4%

**Table 3:** Metagenomic results and PCR test of each case cat.

			Viral Read Numbers																
		Shelter 2									Shelter 3								
	**Cat ID**	**688**	**056**	**160**	**973–975**	**990**	**936**	**723**	**987**	**231**		**787**	**912**	**283**	**178**	**806**	**471**	**594**	**849**
	Total reads (10^6^)	0.76	0.77	1.24	1.11	1.35	1.02	3.16	1.88	0.68		1.29	1.23	1.15	1.41	1.20	1.76	1.21	1.36
Family	*Genus*/Species																		
*Anelloviridae*	*Gyrovirus*																		
	Chicken anemia virus			2															
	Gyrovirus 4														14				
	Gyrovirus 6							2											
	Feline anellovirus						4					6							70
	Other anellovirus					2	44	402					46						
*Parvoviridae*	*Bocaparvovirus*																		
	FeBoV1		34						2	1547									1547
	FeBoV2				63,826		8191	2											
	FeBoV3											1350		12					
	*Chaphamaparvovirus*																		
	Fechavirus			2										8		789	17	1597	6511
	*Copiparvovirus*																		
	Porcine parvovirus 5																2		
	*Dependoparvovirus*																		
	Feline dependoparvovirus														19				8315
	*Protoparvovirus*																		
	Felocell 3 vaccine FPV				112			2086											
*Papillomaviridae*	Dyothetapapillomavirus							7							93				
*Polyomaviridae*	LIPyV										838								
*Caliciviridae*	Felocell 3 vaccine calicivirus	1			2				4										3
PCR test																			
	Fechavirus	+	-	+	−	−	−	−	−	−		−	+	+	−	+	+	+	+
	FeBoV	−	+	−	+	+	+	−	−	+		+	−	+	−	−	−	−	−
	IDEXX diarrhea panel	−	−	−	FPV	+ ^a^	−	ND	ND	ND		+ ^b^	+ ^a^	+ ^c^	ND	+ ^b^	−	+ ^a^	ND
	Rotavirus	−	−	−	−	−	−	ND	−	ND		ND	−	−	−	−	−	−	−

+ ^a^ positive for *C. Perfringens*, + ^b^ positive for *Giardia* and *C. Perfringens*, + ^c^ positive for FECV and *C. Perfringens*; ND: not done.

**Table 4:** Serial fechavirus results from cats tested on multiple dates.

ID	D1	D2	D3	D4	D5	D6	D7	D8	D9	D10	D11	D12
688	+		−									
912	+		−			−	−					
594	+		+	+	+	+	+			+		+
283	−	+	-									

Days (D1–D12) reflect day samples were collected. Days of illness are shaded starting at first sample collection. “+” and “−” means PCR positive or negative for the fechavirus. Illness onset was 10, 2, 2, 3 days before Day 1 for cats #688, 912, 594 and 283, respectively.

## Supplementary Materials

The following are available online at https://www.mdpi.com/1999-4915/12/5/506/s1, Table S1: Clinical signs and case definition ratings for all cats meeting case definition; Table S2: All fecal diagnostic test results for cats meeting case definition.
