# Supplementary material for: Virome of a Feline Outbreak of Diarrhea and Vomiting Includes Bocaviruses and a Novel Chapparvovirus
Source: Viruses. 2020 May 4;12(5):506. doi: 10.3390/v12050506 (PMC7291048; doi:10.3390/v12050506)
Supplement: Supplementary file 1 [file viruses-12-00506-s001.pdf]

Table S1. Clinical signs and case definition ratings for all cats meeting case definition.

Animal ID	Clinical signs onset	Lethargy	Vomiting	Diarrhea	Inappetence	Full resolution of clinical signs	Illness duration in days
182	11/9/18		11/9/18	11/9/18		11/23/18	14
181	11/12/18		11/12/18	11/12/18		11/21/18	9
183	11/12/18		11/12/18	11/12/18		11/21/18	9
853	11/15/18		11/15/18			11/19/18	4
616	11/16/18		11/16/18		12/4/18	11/19/18	3
614	11/16/18		11/16/18	11/16/18	11/16/18	11/21/18	5
688	11/17/18			11/17/18		11/27/18	10
256	11/19/18			11/19/18		11/21/18	2
255	11/19/18			11/19/18		11/21/18	2
160	11/20/18		11/23/18	11/22/18		11/27/18	7
936	11/21/18		11/23/18	11/21/18	11/23/18	12/10/18	19
643	11/23/18	11/25/18	11/23/18	11/24/18	11/25/18	Euth 11/27/18	N/A
973	11/23/18		11/23/18	11/25/18		11/30/18	7
974	11/23/18		11/23/18	11/25/18		11/30/18	7
975	11/23/18		11/23/18	11/25/18		11/30/18	7
080	11/23/18			11/23/18	11/24/18	11/26/18	3
056	11/23/18	12/5/18	11/26/18		11/23/18	11/29/18	6
990	11/23/18		11/23/18	11/23/18		11/28/18	5
596	11/23/18		11/23/18	11/25/18		11/25/18	2
595	11/24/18			11/25/18		11/25/18	1
928	11/27/18	11/28/18	11/27/18		11/28/18	12/1/18	4
929	11/27/18		11/27/18	11/21/18	11/28/18	12/1/18	4
842	11/27/18		11/27/18			11/30/18	3
992	11/27/18			11/27/18		11/30/18	3
694	11/28/18		11/28/18	11/30/18		12/3/18	5
465	11/30/18		12/2/18	11/30/18		12/3/18	3
257	11/30/18		12/2/18	11/30/18		12/3/18	3
989	12/1/18			12/1/18	12/4/18	12/4/18	3
723	12/5/18	12/7/18	12/5/18	12/5/18	12/7/18	12/17/18	12
752	12/6/18		12/6/18	12/9/18		Euth 12/11/18	N/A
987	12/6/18		12/6/18	12/8/18		12/11/18	5
787	12/11/18		12/11/18	12/12/18		12/12/18	1
231	12/20/18		12/20/18	12/21/18		12/24/18	4
400	1/17/19			1/17/19		1/20/19	3
912	1/18/19			1/18/19		1/29/19	11

Animal ID	Clinical signs onset	Lethargy	Vomiting	Diarrhea	Inappetence	Full resolution of clinical signs	Illness duration in days
594	1/20/19	1/20/19		1/23/19	1/20/19	1/25/19	5
283	1/21/19			1/21/19		1/29/19	8
716	1/21/19		1/21/19			1/22/19	1
806	1/22/19			1/22/19		1/28/19	6
911	1/23/19			1/23/19		1/24/19	1
849	1/24/19		1/24/19			1/25/19	1
471	1/24/19			1/24/19		1/25/19	1
178	1/28/19		1/28/19			1/29/19	1

Notes: Orange shading: fechavirus positive
Blue shading: bocavirus positive (3 different species)
Gray shading: fechavirus and bocavirus positive

Table S2: All fecal diagnostic test results for cats meeting case definition

Animal ID	Fecal flotation (in-house)	Fecal flotation with helminth antigen	Multipathogen Diarrhea Panel	Rotavirus PCR	Parvo antigen test	Fechavirus PCR
182*	NEG (11/13)					
181*	NEG (11/13)					
183*	NEG (11/13)					
853					NEG (11/16)	
614					NEG (11/16)	
688		NEG (11/23)	NEG (11/27)	NEG (11/27)		POS (11/27)
160		NEG (11/23)	NEG (11/27)	NEG (11/27)		POS (11/27)
936		NEG (11/23)	NEG (11/27)	NEG (11/27)		NEG (11/27)
233		NEG (11/16)				
973*			POS FPV (11/27)	NEG (11/27)		NEG (11/27)
974*			POS FPV (11/27)	NEG (11/27)		NEG (11/27)
975*			POS FPV (11/27)	NEG (11/27)		NEG (11/27)
080		POS (hookworm/ roundworm) (11/24)				
056			NEG (11/27)	NEG (11/27)		NEG (11/27)
990			POS *C. Perfringens* (11/27)	NEG (11/28)		NEG (11/27)
723		NEG (12/13)			NEG (12/7)	NEG (12/13)
787			POS *Giardia* and *C. Perfringens* (12/12)			NEG (12/12)

**Animal ID**	**Fecal flotation (in-house)**	**Fecal flotation with helminth antigen**	**Multipathogen Diarrhea Panel**	**Rotavirus PCR**	**Parvo antigen test**	**Fechavirus PCR**
231		NEG (01/21)				NEG (01/21)
400		NEG (01/22)				
912		NEG (01/23)	POS *C. Perfringens* (1/20)	NEG (01/20)		POS (01/20)
594			POS *C. Perfringens* (1/23)	NEG (01/23)		POS (01/26)
283			POS *Giardia*, feline enteric coronavirus, *C. Perfringens* (01/25)	NEG (01/25)		POS (01/25)
806			POS *Giardia* and *C. Perfringens* (01/22)	NEG (01/22)		POS (01/22)
849						POS (01/23)
471			NEG (01/24)	NEG (01/24)		POS (01/24)
178						NEG (01/28)
987						NEG (12/6)

*Note: Cats 181, 182, 183 and 973, 974, 975 pooled together (litters of kittens)
NEG = negative result, POS= positive result
Date in parentheses = sample collection date
